# Crystal structure and Hirshfeld surface analysis of the salt 2-iodo­ethyl­ammonium iodide – a possible side product upon synthesis of hybrid perovskites

**DOI:** 10.1107/S205698902401034X

**Published:** 2024-10-31

**Authors:** Hanna R. Petrosova, Dina D. Naumova, Irina A. Golenya, Ildiko Buta, Il’ya A. Gural’skiy

**Affiliations:** a Department of Chemistry, Taras Shevchenko National University of Kyiv, Volodymyrska St. 64, Kyiv 01601, Ukraine; b "Coriolan Dragulescu" Institute of Chemistry, Mihai Viteazu Bvd. 24, Timisoara 300223, Romania; Vienna University of Technology, Austria

**Keywords:** crystal structure, organic cation, ammonium salt, iodide, hydrogen bonding

## Abstract

The title organic–inorganic hybrid salt, C_2_H_7_IN^+^·I^−^, is isotypic with its bromine analog, C_2_H_7_BrN^+^·Br^−^ [Semenikhin *et al.* (2024 ▸). *Acta Cryst.* E**80**, 738–741]. Its asymmetric unit consists of one 2-iodo­ethyl­ammonium cation and one iodide anion. The NH_3_^+^ group of the organic cation forms weak hydrogen bonds with four neighboring iodide anions, leading to the formation of supra­molecular layers propagating parallel to the *bc* plane. Hirshfeld surface analysis reveals that the most important contribution to the crystal packing is from N—H⋯I inter­actions (63.8%). The crystal under investigation was twinned by a 180° rotation around [001].

## Abstract 2

The organic 2-iodo­ethyl­ammonium cation forms N—H⋯I hydrogen bonds with iodide anions, forming supra­molecular layers.

## 1. Chemical context

Hybrid organic–inorganic perovskites are known for their inter­esting semiconducting and optical properties, which allow their application in photovoltaic and optoelectronic devices (Younis *et al.*, 2021 ▸). At the same time, hybrid perovskites create an equally fascinating background for fundamental studies by forming numerous structural motifs of different periodicities.

Even though the structure type *perovskite* usually refers to inorganic compounds with composition *ABX*_3_ (Li *et al.*, 2017 ▸), recent developments in this field led to ‘hybrid organic-inorganic perovskites’, which contain discrete or fused [*BX*_6_]^*n*−^ octa­hedral building units of inorganic nature, the charge of which is compensated by organic cations. The corresponding octa­hedra can be connected to each other in various ways, resulting in frameworks with different periodicity (Han *et al.*, 2021 ▸).

However, an important issue that demands extra caution upon work with hybrid perovskites is their stability. These materials are very sensitive to water vapor, which can cause their immediate degradation to different products including inorganic salts such as *BX*_2_ and organic salts *AX*.
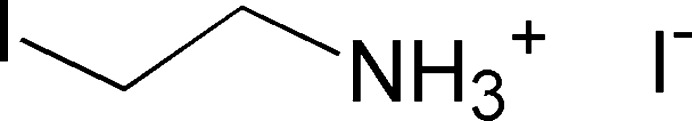


Here we report on synthesis and crystal structure of the organic–inorganic hybrid salt 2-iodo­ethyl­ammonim iodide, C_2_H_7_IN^+^·I. The 2-iodo­ethyl­ammonim cation has previously been incorporated into some hybrid perovskites with layered arrangements (Xue *et al.*, 2023 ▸; Skorokhod *et al.*, 2023 ▸). In addition, 2-iodo­ethyl­ammonim can be formed as a result of aziridine ring-opening reaction upon synthesis of aziridinium perovskites (Kucheriv *et al.*, 2023 ▸; Petrosova *et al.*, 2022 ▸). Therefore, the reported structural data of the title compound are valuable for phase analysis, since such a phase can be a side product in the synthesis of hybrid perovskites with 2-iodo­ethyl­ammonium or aziridinium cations.

## 2. Structural commentary

**Figure 1:**
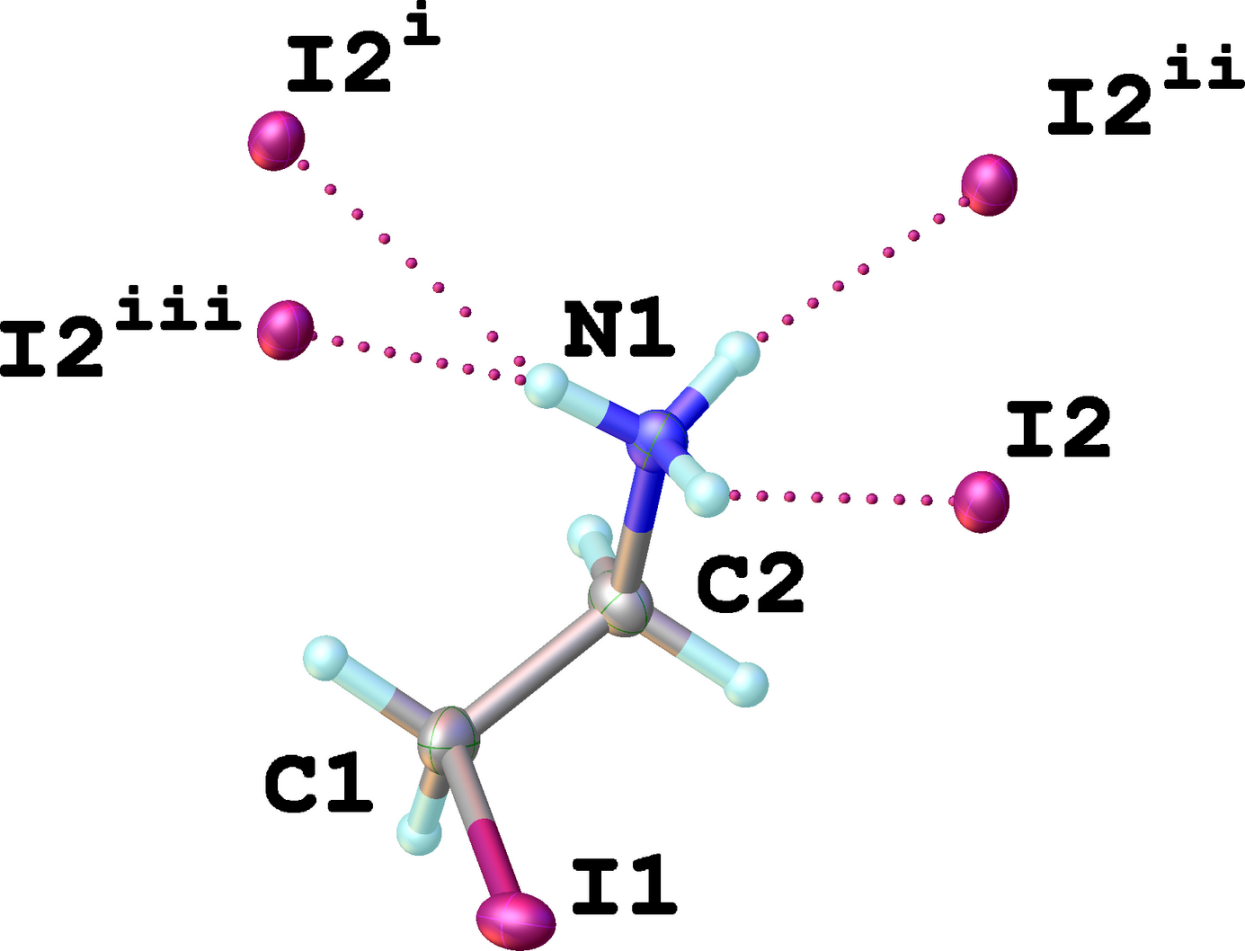
The expanded asymmetric unit of the title compound showing hydrogen bonds between the organic cation and iodide anions (dotted lines). Displacement ellipsoids are drawn at the 50% probability level; symmetry codes refer to Table 1 ▸.

The asymmetric unit consists of one organic 2-iodo­ethyl­ammonium cation and an iodide counter-ion (Fig. 1 ▸). The I1—C1 bond length is 2.170 (10) Å, that of C1—C2 is 1.497 (14) Å and of C2—N1 is 1.514 (12) Å. The torsion angle I1—C1—C2—N1 is −65.8 (9)°, indicating that the organic cation adopts a synclinal conformation. It is worth noting, that the organic cation IC_2_H_4_NH_3_^+^ has previously been reported in other crystal structures, but only as a part of hybrid organic–inorganic perovskites (see *Database survey*). The analysis of these structures reveals that this cation can adopt both synclinal and anti­periplanar conformations inside the inorganic frameworks depending on the strength and orientation of the hydrogen bonds formed (Xue *et al.*, 2023 ▸).

## 3. Supra­molecular features

**Figure 2:**
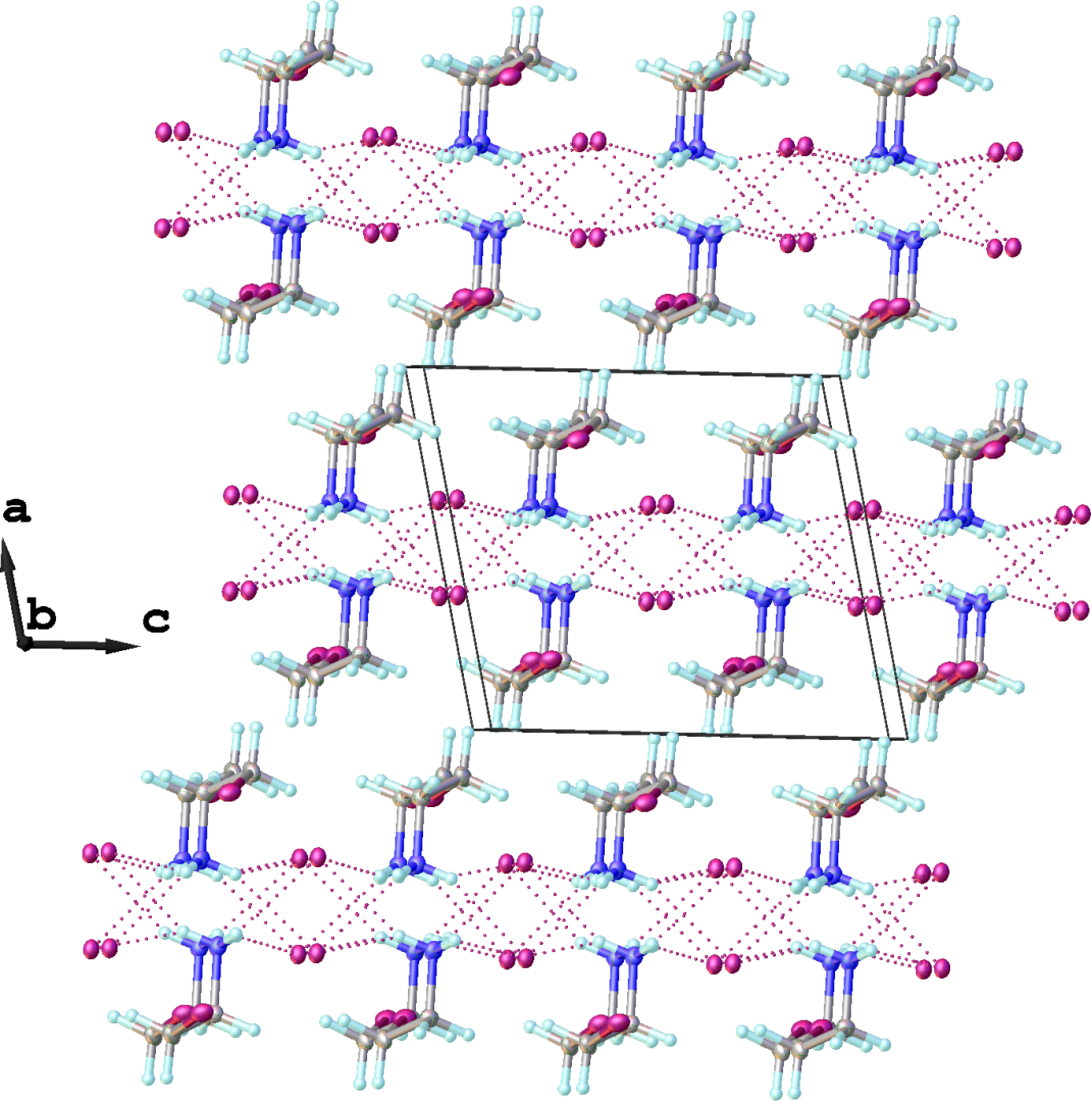
Crystal packing of the title compound in a view approximately along [010] showing infinite supra­molecular layers propagating parallel to the *bc* plane.

**Figure 3:**
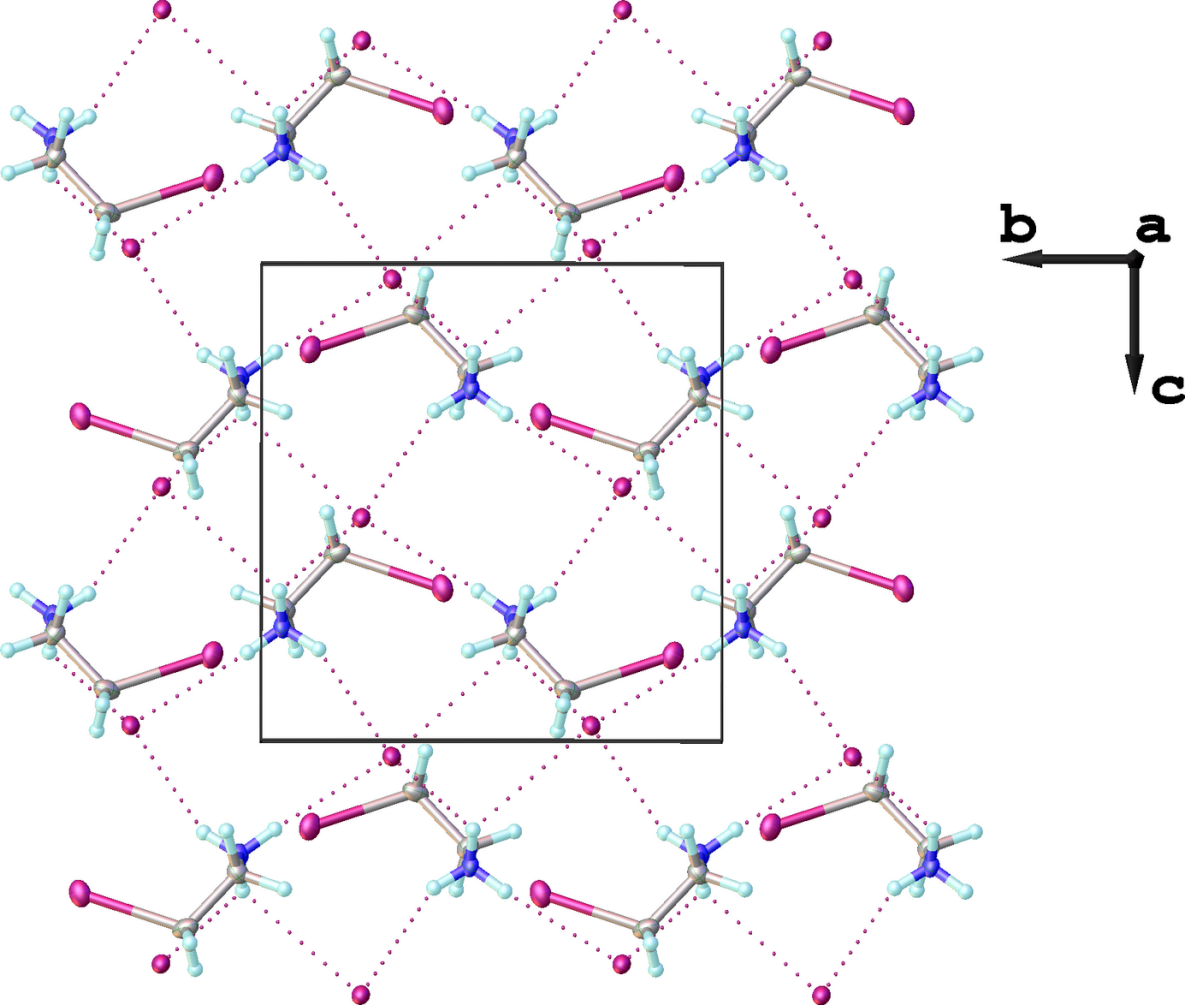
A single supra­molecular layer viewed along [100].

Each 2-iodo­ethyl­ammonium cation is connected to four different iodide anions through weak inter­molecular N—H⋯I inter­actions (Fig. 1 ▸). Simultaneously, each iodide anion forms hydrogen bonds with four neighboring –NH_3_^+^ groups of 2-iodo­ethyl­ammonium cations, forming infinite supra­molecular layers propagating parallel to the *bc* plane (Fig. 2 ▸). A view along the *a* axis of a single supra­molecular layer is given in Fig. 3 ▸. Numerical parameters of the hydrogen-bonding inter­actions are compiled in Table 1 ▸.

## 4. Hirshfeld surface analysis

**Figure 4:**
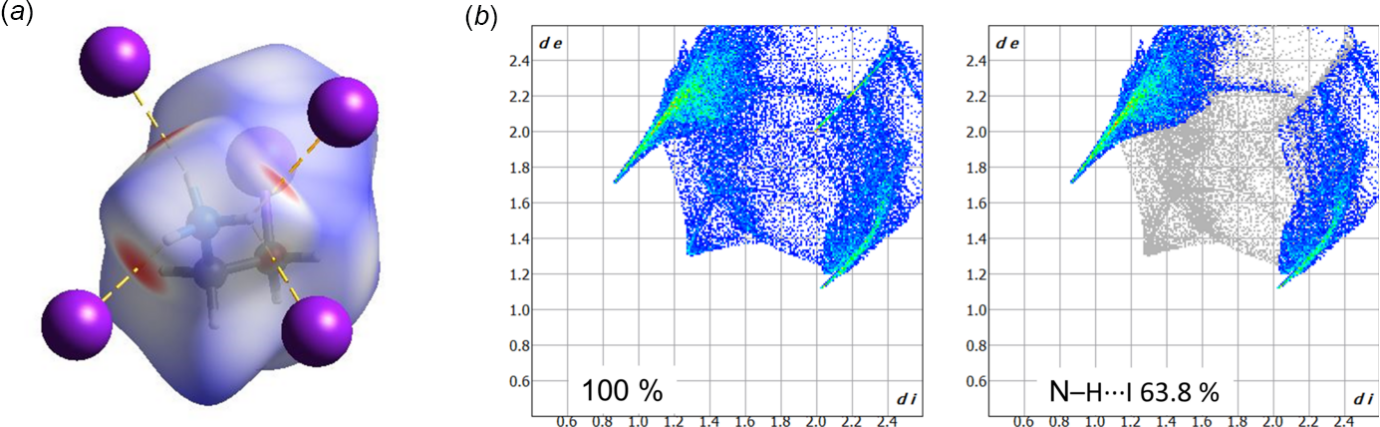
(*a*) Hirshfeld surface of the 2-iodo­ethyl­ammonium cation plotted over *d*_norm_, showing the strongest inter­actions with I^−^ anions (in red); (*b*) the two-dimensional fingerprint plots for 2-iodo­ethyl­ammonium iodide (left) and delineated into N—H⋯I contacts (right).

Hirshfeld surface analysis (Hirshfeld, 1977 ▸; Spackman & Jayatilaka, 2009 ▸) was used to visualize and qu­antify inter­molecular inter­actions in 2-iodo­ethyl­ammonium iodide using *CrystalExplorer* (Spackman *et al.*, 2021 ▸). The Hirshfeld surface plotted over *d*_norm_ and the two-dimensional fingerprint plots are given in Fig. 4 ▸. The surface shows the four N—H⋯I contacts described above as regions colored in red (Fig. 4 ▸*a*), where the color code denotes contacts with distances equal to the sum of the van der Waals radii as white, while those with shorter and longer distances are represented in red and blue, respectively. The two-dimensional fingerprint plots show that the most important inter­action found in the structure is represented by N—H⋯I contacts, which account for 63.8% of all contacts observed in the crystal structure (Fig. 4 ▸*b*). The residual contributions originate from H⋯H inter­actions.

## 5. Database survey

A search in the Cambridge Crystallographic Database (CSD, version 5.45, update of September 2024; Groom *et al.*. 2016 ▸) for the 2-iodo­ethyl­ammonium cation revealed the following structures, which all are based on perovskite-type inorganic anions: JIGYEH, JIGYIL, JIGYUX (Skorokhod *et al.*, 2023 ▸); SIWHIQ, TEYMIU (Sourisseau *et al.*, 2007 ▸), TEGROQ (Song *et al.*, 2022 ▸). The title compound is isotypic with 2-bromo­ethyl­ammonium bromide (ZOTHAV; Semenikhin *et al.*, 2024 ▸).

## 6. Synthesis and crystallization

All reagents were purchased from UkrOrgSynthez Ltd. and used as received. Aziridine (50 µl) was added to aqueous HI (57% w/w, 300 µl) and was left to crystallize at room temperature. Colorless crystals were harvested after one day and protected under Paratone^® ^oil.

## 7. Refinement

Crystal data, data collection and structure refinement details are summarized in Table 2 ▸. The crystal under investigation was twinned by a 180° rotation around [001] and the intensity data processed into a HKLF5-type file; the twin components refined to a ratio of 0.617 (2):0.383 (2). Hydrogen atoms were placed at calculated positions with *U*_iso_(H) = 1.2*U*_eq_(C) and *U*_iso_(H) = 1.5*U*_eq_(N).

## Supplementary Material

Crystal structure: contains datablock(s) I. DOI: 10.1107/S205698902401034X/wm5738sup1.cif

Structure factors: contains datablock(s) I. DOI: 10.1107/S205698902401034X/wm5738Isup2.hkl

Supporting information file. DOI: 10.1107/S205698902401034X/wm5738Isup3.cml

CCDC reference: 2393092

Additional supporting information:  crystallographic information; 3D view; checkCIF report

## Figures and Tables

**Table 1:** Hydrogen-bond geometry (Å, °)

*D*—H⋯*A*	*D*—H	H⋯*A*	*D*⋯*A*	*D*—H⋯*A*
N1—H1*A*⋯I2^i^	0.84	2.74	3.517 (7)	155
N1—H1*B*⋯I2	0.84	2.85	3.618 (8)	153
N1—H1*C*⋯I2^ii^	0.84	2.96	3.627 (8)	139
N1—H1*C*⋯I2^iii^	0.84	3.04	3.600 (8)	127

Symmetry codes: (i) 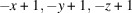; (ii) 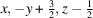; (iii) 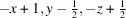.

**Table 2:** Experimental details

Crystal data	
Chemical formula	C_2_H_7_IN^+^·I^−^
*M*_r_	298.89
Crystal system, space group	Monoclinic, *P*2_1_/*c*
Temperature (K)	100
*a*, *b*, *c* (Å)	8.3073 (7), 8.8800 (6), 9.3838 (7)
β (°)	102.004 (7)
*V* (Å^3^)	677.10 (9)
*Z*	4
Radiation type	Mo *K*α
μ (mm^−1^)	9.16
Crystal size (mm)	0.31 × 0.15 × 0.07

Data collection	
Diffractometer	XtaLAB Synergy, Dualflex, HyPix
Absorption correction	Analytical (*CrysAlis PRO*; Rigaku OD, 2023 ▸)
*T*_min_, *T*_max_	0.157, 0.573
No. of measured, independent and observed [*I* > 2σ(*I*)] reflections	2744, 2744, 2535
(sin θ/λ)_max_ (Å^−1^)	0.712

Refinement	
*R*[*F*^2^ > 2σ(*F*^2^)], *wR*(*F*^2^), *S*	0.051, 0.159, 1.12
No. of reflections	2744
No. of parameters	49
H-atom treatment	H atoms treated by a mixture of independent and constrained refinement
Δρ_max_, Δρ_min_ (e Å^−3^)	2.07, −1.68

Computer programs: *CrysAlis PRO* (Rigaku OD, 2023 ▸), *SHELXT* (Sheldrick, 2015*a* ▸), *SHELXL* (Sheldrick, 2015*b* ▸), *OLEX2* (Dolomanov *et al.*, 2009 ▸) and *publCIF* (Westrip, 2010 ▸).

## supplementary crystallographic information

### 2-Iodoethylammonium iodide Crystal data

**Table:**

C_2_H_7_IN^+^·I^−^	*F*(000) = 528
*M**_r_* = 298.89	*D*_x_ = 2.932 Mg m^−^^3^
Monoclinic, *P*2_1_/*c*	Mo *K*α radiation, λ = 0.71073 Å
*a* = 8.3073 (7) Å	Cell parameters from 3490 reflections
*b* = 8.8800 (6) Å	θ = 3.2–30.3°
*c* = 9.3838 (7) Å	µ = 9.16 mm^−^^1^
β = 102.004 (7)°	*T* = 100 K
*V* = 677.10 (9) Å^3^	Plate, clear intense colourless
*Z* = 4	0.31 × 0.14 × 0.07 mm

### 2-Iodoethylammonium iodide Data collection

**Table:**

XtaLAB Synergy, Dualflex, HyPix diffractometer	2744 independent reflections
Detector resolution: 10.0000 pixels mm^-1^	2535 reflections with *I* > 2σ(*I*)
ω scans	θ_max_ = 30.4°, θ_min_ = 2.5°
Absorption correction: analytical (CrysAlisPro; Rigaku OD, 2023)	*h* = −11→10
*T*_min_ = 0.157, *T*_max_ = 0.573	*k* = −11→11
2744 measured reflections	*l* = −10→12

### 2-Iodoethylammonium iodide Refinement

**Table:**

Refinement on *F*^2^	0 restraints
Least-squares matrix: full	Hydrogen site location: inferred from neighbouring sites
*R*[*F*^2^ > 2σ(*F*^2^)] = 0.051	H atoms treated by a mixture of independent and constrained refinement
*wR*(*F*^2^) = 0.159	*w* = 1/[σ^2^(*F*_o_^2^) + (0.1098*P*)^2^ + 4.1089*P*] where *P* = (*F*_o_^2^ + 2*F*_c_^2^)/3
*S* = 1.12	(Δ/σ)_max_ < 0.001
2744 reflections	Δρ_max_ = 2.07 e Å^−^^3^
49 parameters	Δρ_min_ = −1.68 e Å^−^^3^

### 2-Iodoethylammonium iodide Special details

**Table:**

Geometry. All esds (except the esd in the dihedral angle between two l.s. planes) are estimated using the full covariance matrix. The cell esds are taken into account individually in the estimation of esds in distances, angles and torsion angles; correlations between esds in cell parameters are only used when they are defined by crystal symmetry. An approximate (isotropic) treatment of cell esds is used for estimating esds involving l.s. planes.
Refinement. Refined as a 2-component twin.

### 2-Iodoethylammonium iodide Fractional atomic coordinates and isotropic or equivalent isotropic displacement parameters (Å^2^)

**Table:**

	*x*	*y*	*z*	*U*_iso_*/*U*_eq_	
I2	0.63381 (7)	0.78334 (6)	0.53313 (6)	0.0165 (2)	
I1	0.19546 (8)	0.89307 (7)	0.17832 (7)	0.0224 (2)	
N1	0.3819 (10)	0.5459 (8)	0.2618 (8)	0.0176 (15)	
H1A	0.412 (3)	0.471 (7)	0.314 (7)	0.026*	
H1B	0.417 (3)	0.624 (7)	0.307 (8)	0.026*	
H1C	0.419 (3)	0.540 (8)	0.186 (6)	0.026*	
C2	0.1958 (12)	0.5506 (11)	0.2219 (10)	0.0200 (18)	
H2A	0.153639	0.449803	0.187631	0.024*	
H2B	0.152455	0.575673	0.309573	0.024*	
C1	0.1349 (13)	0.6643 (12)	0.1053 (10)	0.024 (2)	
H1D	0.013934	0.654792	0.073950	0.028*	
H1E	0.183992	0.642845	0.019956	0.028*	

### 2-Iodoethylammonium iodide Atomic displacement parameters (Å^2^)

**Table:**

	*U*^11^	*U*^22^	*U*^33^	*U*^12^	*U*^13^	*U*^23^
I2	0.0214 (4)	0.0135 (4)	0.0150 (4)	−0.00033 (17)	0.0051 (3)	0.00083 (17)
I1	0.0189 (4)	0.0192 (4)	0.0301 (4)	0.0035 (2)	0.0068 (3)	0.0037 (2)
N1	0.022 (4)	0.013 (3)	0.019 (4)	−0.002 (3)	0.006 (3)	0.001 (3)
C2	0.020 (4)	0.020 (5)	0.020 (5)	−0.001 (3)	0.003 (4)	0.003 (3)
C1	0.024 (5)	0.029 (5)	0.017 (4)	0.004 (4)	0.001 (4)	−0.005 (4)

### 2-Iodoethylammonium iodide Geometric parameters (Å, º)

**Table:**

I1—C1	2.170 (10)	C2—H2A	0.9900
N1—H1A	0.84 (6)	C2—H2B	0.9900
N1—H1B	0.84 (6)	C2—C1	1.497 (14)
N1—H1C	0.84 (6)	C1—H1D	0.9900
N1—C2	1.514 (12)	C1—H1E	0.9900
			
H1A—N1—H1B	109.5	C1—C2—N1	111.9 (8)
H1A—N1—H1C	109.5	C1—C2—H2A	109.2
H1B—N1—H1C	109.5	C1—C2—H2B	109.2
C2—N1—H1A	109.5	I1—C1—H1D	109.1
C2—N1—H1B	109.5	I1—C1—H1E	109.1
C2—N1—H1C	109.5	C2—C1—I1	112.3 (6)
N1—C2—H2A	109.2	C2—C1—H1D	109.1
N1—C2—H2B	109.2	C2—C1—H1E	109.1
H2A—C2—H2B	107.9	H1D—C1—H1E	107.9
			
N1—C2—C1—I1	−65.8 (9)		

### 2-Iodoethylammonium iodide Hydrogen-bond geometry (Å, º)

**Table:**

*D*—H···*A*	*D*—H	H···*A*	*D*···*A*	*D*—H···*A*
N1—H1*A*···I2^i^	0.84	2.74	3.517 (7)	155
N1—H1*B*···I2	0.84	2.85	3.618 (8)	153
N1—H1*C*···I2^ii^	0.84	2.96	3.627 (8)	139
N1—H1*C*···I2^iii^	0.84	3.04	3.600 (8)	127

Symmetry codes: (i) −*x*+1, −*y*+1, −*z*+1; (ii) *x*, −*y*+3/2, *z*−1/2; (iii) −*x*+1, *y*−1/2, −*z*+1/2.
